# Towards the Disease Biomarker in an Individual Patient Using Statistical Health Monitoring

**DOI:** 10.1371/journal.pone.0092452

**Published:** 2014-04-01

**Authors:** Jasper Engel, Lionel Blanchet, Udo F. H. Engelke, Ron A. Wevers, Lutgarde M. C. Buydens

**Affiliations:** 1 Radboud University Nijmegen, Institute for Molecules and Materials, Nijmegen, the Netherlands; 2 Laboratory of Genetic Endocrine and Metabolic Diseases at the Department of Laboratory Medicine, Radboud University Medical Centre, Nijmegen, the Netherlands; 3 Department of Biochemistry, Nijmegen Centre for Molecular Life Sciences, Radboud University Medical Centre, Nijmegen, the Netherlands; Mayo Clinic, United States of America

## Abstract

In metabolomics, identification of complex diseases is often based on application of (multivariate) statistical techniques to the data. Commonly, each disease requires its own specific diagnostic model, separating healthy and diseased individuals, which is not very practical in a diagnostic setting. Additionally, for orphan diseases such models cannot be constructed due to a lack of available data. An alternative approach adapted from industrial process control is proposed in this study: statistical health monitoring (SHM). In SHM the metabolic profile of an individual is compared to that of healthy people in a multivariate manner. Abnormal metabolite concentrations, or abnormal patterns of concentrations, are indicated by the method. Subsequently, this biomarker can be used for diagnosis. A tremendous advantage here is that only data of healthy people is required to construct the model. The method is applicable in current–population based –clinical practice as well as in personalized health applications. In this study, SHM was successfully applied for diagnosis of several orphan diseases as well as detection of metabotypic abnormalities related to diet and drug intake.

## Introduction

Metabolomics is becoming increasingly important in a whole range of healthcare-related fields such as disease prevention, diagnosis and intervention, and studies of the impact of diet and nutrition on various forms of illness [1]–[3]. In such studies, the metabolic phenotype or metabotype of individuals is studied. The metabotype is a characteristic metabolite profile that depends on the interplay between genes and environmental factors such as diet, lifestyle, gut microbial composition, and – in patients – medication. This profile varies greatly between individuals and populations. Therefore, metabotyping has applications in population-based and personalized medicine [1],[4]. For example, various (subtle) abnormalities in the metabotype have been related to cancer states, diabetes, cardiovascular diseases, neurological diseases and inborn errors of metabolism (IEM) [1], [3], [5], [6].

Commonly, the metabotype of an individual is measured from easily accessible biofluids such as urine or serum, or more seldom from tissue [2]. Typically, untargeted metabolomics techniques such as ^1^H nuclear magnetic resonance (NMR) spectroscopy or mass-spectrometric methods are used for this purpose [2], [7], [8]. These techniques can measure a wide range of metabolites simultaneously and generate a multivariate profile of metabolites present in the sample. Due to the complex nature of the metabolome in biofluids multivariate data analysis is often required to interpret the acquired data and detect metabolic abnormalities. Most studies deal with classification problems such as disease diagnosis (healthy versus a specific disease) [3], [9]. During data analysis, such problems are commonly tackled as a *two-class* or a *one-class* problem.

Two-class classification strategies aim to model the metabolic differences *between* groups of healthy and diseased individuals. These differences are grouped in a metabolic pattern or biomarker representing the abnormalities related to the disease. Typically used methods for two-class classification are orthogonal projection to latent structure (OPLS) and linear discriminant analysis (LDA) [9]. More challenging problems are generally tackled using non-linear approaches such as SVM, K-PLS or Random Forests [10], [11]. In contrast, one-class classification methods focus on the similarities that are encountered *within* the diseased group. This results in a characterization of the expected metabotypes for a specific disease (e.g. an average metabotype and expected metabolic variation). Here, the most commonly used technique is SIMCA [9]. For both classification strategies, a diagnosis is made by matching the metabotype of a patient against the result of the model, being this biomarker or expected metabotype.

Both strategies focus on groups of patients with one specific disease. This might be impractical in a clinical setting for three reasons. First, it is not realistic to construct a statistically valid model for rare or orphan diseases. Such diseases are defined in the United States as any disease that affects fewer than 200000 individuals, and in the European Community as any disease that affects fewer than 5 in 10000 individuals [12]. Some rare diseases have less than a dozen known cases. In other words, the number of potential patients to base the model on is too low. Secondly, even if orphan diseases are ignored, each disease requires its own specific model. Thirdly, unknown metabolic perturbations, for instance caused by unknown diseases, may not be detected or falsely interpreted.

Interestingly, similar problems are encountered when monitoring industrial processes. Analogous to disease diagnosis one wants to know whether or not the process is in-control (healthy); if not, a known or unknown rare event (a disease) has occurred that may affect product quality. So-called statistical process control (SPC) techniques have been developed to detect all of these events as early as possible [13]. Due to the success of SPC, we propose here to adapt these strategies and apply the method on metabolome profiles of body fluids with the aim of diagnosing the disease of a patient. This provides a new tool for diagnostic support: statistical health monitoring (SHM).

In SHM, the so-called normal operating conditions (NOC) of healthy people are defined. NOC is a term that is often used in SPCA. In this case it basically means that a one-class classifier is used to model the expected metabotypes of healthy individuals. The NOC should therefore represent the average metabotype of a population and the inherent (normal) variation present in this population e.g. due to difference in life style. Next, the metabotype of a patient is compared to NOC. Deviations from NOC such as abnormal metabolite concentrations or abnormal patterns of concentrations are indicated by the method. Subsequently, this information – a (disease) biomarker for this individual patient – can be used for diagnosis. The fact that only data of healthy people is required to construct the SHM model is a tremendous advantage of this approach. Because of this, SHM is not disease specific and can be used for diagnosis of rare diseases.

As a case study we applied SHM for diagnosis of a family of orphan diseases, namely inborn errors of metabolism. IEM comprise a substantial group of rare genetic diseases that can be diagnosed by NMR spectroscopy in combination with visual inspection of the data [5]. Because of the complex structure of the spectra this can be quite a challenge. Moreover, such an approach is extremely time-consuming and quite subjective. The proposed SHM approach may make the diagnosis of IEM easier and objective. Additionally, it will be shown that, depending on how the NOC are defined, SHM can also detect metabolic abnormalities related to diet and medication.

The next section will outline the concept of SHM and mathematic background. In the remaining sections the properties of SHM are discussed based on application of SHM to the case study example involving IEM.

## Theory of Statistical Health Monitoring

In SHM the metabotype of an individual is compared to that of healthy people in a multivariate manner. Abnormal metabolite concentrations, or abnormal patterns of concentrations, are indicated by the method. This is achieved in two steps. In this first step – detection of abnormal metabotypes – the metabotype of an individual is matched against NOC and marked as normal or possibly abnormal. The abnormal metabolites are identified in a second step.

### Detection of abnormal metabotypes

The first step in SHM is to select samples that represent the NOC of healthy humans well. From now on we will refer to these samples as normal or NOC samples. The choice of NOC samples should reflect the goal of the SHM analysis. For example, if the goal is purely to detect abnormalities related to disease, the NOC set can include healthy individuals who recently took medication. However, if one also wants to detect abnormal metabolites related to drugs, these individuals should not be included. Additionally, the demographics of the NOC samples and the expected patients should be as similar as possible. For example, if a patient has a completely different lifestyle compared to the NOC samples, many metabolites may falsely be marked as abnormal. However, if the demographics are too loosely specified, the limit of detection of the SHM model will be negatively affected. We will further elaborate on this important aspect in the discussion section.

The NOC samples are stored in data table (**X**_h_). Each row in **X**_h_ contains the metabotype information from one healthy individual. Each column corresponds to a measured feature, e.g. a chemical shift value in an NMR spectrum. The data is centred to zero mean before starting the statistical analysis. Often it is also useful to scale the data – e.g. to unit variance – to ensure that each feature has equal chance to influence the model.

Principal component analysis (PCA) is used to describe the NOC data [13]: 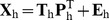
(1)


Here, **T**_h_ **P**_h_^T^ is the part of the model that describes the structural metabolic variation between the NOC samples, while matrix **E**_h_ only contains residuals or non-structural variation. **T**_h_ describes the systematic metabolic differences or variation between the NOC samples. The columns in **P**_h_, or factors, are the actual model. The factors are descriptors that indicate in which measured features the systematic differences occur. A property of the factors is that they are ordered by importance: the first “explains” most variation, followed by the second, etc. At some point the remaining factors only describe noise. These factors are not included in the model.

To determine whether someone is possibly diseased, the metabotype information from this individual (**x**_new_) is evaluated using the constructed model: 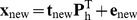
(2)


Note that **t**_new_ **P**_h_^T^ describes which part of the individual's metabotype is in accordance with the metabotypes that are expected for NOC samples. If an individual is similar to the normal samples, this prediction should capture his/her complete metabotype. In this case, the error **e**_new_ should be small and fall in the range of the error of the NOC samples. Therefore, abnormal metabotypes can be detected by inspection of the size of **e**_new_. In industrial process control, the so-called *Q*-statistic is used for this purpose [13]:
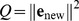
(3)


A sample with a high *Q*-value corresponds to a metabotype that either contains abnormal metabolite(s) or abnormal metabolite concentrations that break the normal between-metabolites correlation pattern. The metabotype of an individual is marked as abnormal if the value for *Q* exceeds the significance limit given by *Q_α_* [14]: 
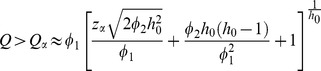
(4)where the parameters of the approximation are defined as 
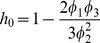
(5)

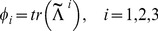
(6)

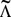
 is the covariance matrix of **E**_h_, and *z_α_* is the standardized normal variable with (1−*α*) confidence limit, having the same sign as *h_0_* [14].

To summarize, for a measured metabotype (**x**_new_) the PCA model (**t**_new_ **P**_h_^T^) is used to predict what this metabotype would look like if the individual was an NOC sample. The larger the difference between predicted and measured metabotype (**e**_new_) the more likely the metabotype is to be abnormal. The size of this difference is expressed via the *Q*-statistic. An example of SHM when monitoring 2 metabolites is presented in File S1.

### Identification of abnormal metabolites

The second step in SHM is to detect the abnormal metabolites that caused the deviation from NOC. A clinical practitioner can use this information for example for disease diagnosis, possibly via a database search.

Since the *Q*-statistic should detect all deviations from NOC, the contribution of measured features to this statistic should be investigated. For this purpose, the value for *Q* is decomposed into per feature contributions. Here, we used partial decomposition [15]: 
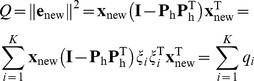
(7)where **I** is the identity matrix and **ξ** *_i_* is the *i* th column of the identity matrix. Index *i* and *K* indicate a specific feature and the total number of measured features, respectively. The contribution of the measured value in feature *i* to *Q* equals *q_i_*. High values indicate abnormal behaviour of this particular feature. However, the contribution of each feature in the NOC data should be taken into account as well: a large contribution of a feature becomes less meaningful if such contribution values were also observed for the NOC samples. Therefore, all contributions were studied relatively to the variance of the NOC residuals **E**_h_ [15]: 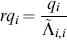
(8)


Where 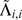 indicates the element in the *i* th row and column of 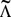.

The set of relative contributions for all features will be referred to as a personal health profile or personal biomarker.

## Method and Materials

### Data

To assess the value of SHM for disease diagnosis, a set of urine samples of 193 healthy children and a set of 24 patients was measured using proton NMR spectroscopy. Eighteen patients were known to suffer from one of seven different IEM. For the other six patients, no IEM was diagnosed, but their metabotypes contained commonly prescribed drugs such as depakine and sabril. More details regarding the healthy and patient samples are specified in Tables 1 and 2, respectively. Note that a subject had to be between 4–12 years old to participate in this study and be of Dutch ancestry. An equal amount of males and females were selected.No other selection criteria such as lifestyle and diet were imposed.

**Table 1:** Abnormal compounds present in urine 1D ^1^H-NMR spectra from the healthy individuals.

Compound	CS (ppm); multiplicity	Origin
Acesulfame	2.11d; 5.67q	Artificial sweetener
Acetaminophen	2.15s; 6.90d; 7.25d	Paracetamol
A-glucuronide*	2.16s; 5.12d; 7.13d; 7.34d	Paracetamol
A-*L*-cysteinyl*	2.15s; 6.99d; 7.51d	Paracetamol
A-*N*-acetyl-L-cysteinyl*	1.84s; 2.14s; 6.93d; 7.42d	Paracetamol
A-Sulphate*	2.17s; 7.45d; 7.31d	Paracetamol
Cyclamate	1.53–2.06m	Artificial sweetener
Mannitol	3.6–3.8v	Sweetener
*N*-Methylhydantoin	2.92s; 4.08s	Bacteria
TMA-oxide	3.54s	Fish meal

The metabolites were identified by comparison of the abnormal resonances to a database of NMR spectra of model compounds [5]. In cases where the overlap of resonances in the 1D spectrum was quite severe, 2D COSY NMR experiments were used to provide additional information and confirm that the metabolite identification based on the 1D spectrum was correct.

*Spectrum not completed interpreted; s = singlet; d = doublet; t = triplet; q = quartet; m = multiplet; v = various multipets.

A =  Acetaminophen; TMA =  Trimethylamine.

**Table 2:** Abnormal compounds present in urine 1D ^1^H-NMR spectra from the patients.

Compound	CS (ppm); multiplicity	IEM/Orign
Arginine	1.69m; 1.92m; 3.24q; 3.85t	Cystinuria
4-Amino-5-hexenoic acid	1.94m; 2.08m; 2.46m; 5.47m; 5.80m	Medication: Sabril
Dihydroxycholenic acid	0.67s; 0.80–0.94v	3β-Hydroxy-Δ^5^-C_27_-steroid dehydrogenase deficiency
Formiminoglutamic acid	2.00–2.22v; 2.47t	Formiminotransferase deficiency
Homogentisic acid	3.64s; 6.78m	Alkaptonuria
Hydantoin-5-propionic acid	2.00–2.22v; 2.51t	Formiminotransferase deficiency
3-Hydroxyisovaleric acid	1.33s; 2.55s	Isovaleric aciduria 3MCC-deficiency
Isovalerylglycine	0.94d; 2.02m; 2.18d; 3.94d	Isovaleric aciduria
Lysine	1.47m; 1.72m; 1.92m; 3.01t; 3.77t	Cystinuria
3-Methylcrotonylglycine	1.86d; 2.03d; 3.97d; 5.78m	3MCC-deficiency
2-oxo-1-pyrrolidine acetamide	2.10m; 2.48t; 3.52t; 4.01s	Medication: Piracetam
5-Oxoproline	2.20m; 2.43m; 2.55m; 4.36m	5-Oxoprolinuria
Taurine	3.27t; 3.43t(wide due to exchange)	Unknown; possibly nutrition
Trihydroxycholenic acid	0.73s; 0.80–0.94v	3β-Hydroxy-Δ^5^-C_27_-steroid dehydrogenase deficiency
Valproic acid	0.88t; 1.30m; 1.50m; 2.44m	Medication: Depakine

The metabolites were identified by comparison of the abnormal resonances to a database of NMR spectra of model compounds [5]. In cases where the overlap of resonances in the 1D spectrum was quite severe, 2D COSY NMR experiments were used to provide additional information and confirm that the metabolite identification based on the 1D spectrum was correct.

*Spectrum not completed interpreted; s = singlet; d = doublet; t = triplet; q = quartet; m = multiplet; v = various multipets.

3MCC = 3-Methylcrotonyl CoA carboxylase.

The urine samples were centrifuged before analysis. A volume of 70 μl of a 20.2 mmol/l trimethylsilyl-2,2,3,3-tetradeuteriumpropionic acid (TSP, sodium salt; Aldrich) ^2^H_2_O solution was added to 700 μl of urine as a chemical shift reference (δ = 0.00) and as a lock signal. The pH of the urine was adjusted to 2.50±0.05 with HCl. Finally, 650 μl of the sample was placed into a 5-mm NMR tube (Wilmad Royal Imperial; Wilmad LabGlass, USA).

1H NMR spectra were °btained using a Bruker 500 MHz spectrometer (pulse angle 90°, delay time 4 s, number of scans 256, temperature 298 K). The water resonance was suppressed by gated irradiation centred on the water frequency. Shimming was performed automatically on each sample prior to the data acquisition using the TopShim method from Bruker BioSpin.The phase and baseline were corrected manually.

The regions 0.2–4.7 ppm and 5.0–10.0 ppm were selected for further analysis in Matlab 7.14 (Mathworks, Natick, Massachusetts, U.S.A.). Next, the urine profiles were normalized to the creatinine signal to correct for dilution effects. Equidistant binning with a bin size of 0.04 ppm was used to reduce the dimension of the normalized data from 30888 to 246 variables. Finally, the data was centred to zero mean and scaled to unit variance.

For some samples (see below), 2D COSY NMR spectra were also recorded for extra spectral information. The spectra were recorded at 500 MHz using 4 k data points in F2 and a spectral with of 6002 Hz. For all samples, 256 increments and 16 scans per increment were used. The TR was 2 s, during which the water resonance was presaturated. Prior to Fourier transformation, a since function was applied in both time domains.

### Ethics statement

The medical ethical committee of the Radboud University Medical Centre in Nijmegen, The Netherlands, approved the study protocol. Informed verbal parental consent was obtained for all volunteers. At that time consultation of the medical ethical committee of the Radboud University Medical Centre in Nijmegen, The Netherlands resulted in the advice to ask the collaboration of the parents/caretakers of the children via an information letter explicitly stating that their collaboration was on a voluntary basis and that all samples would be fully anonymized and that all samples would be destroyed at the latest 5 years after the sample collection.

### SHM model construction and validation

First, the raw NMR spectra of the 193 healthy individuals were extensively screened by an experienced clinical practitioner to rule out any abnormal metabolic patterns in these samples. Seventeen samples with abnormal patterns related to dietary influences and drug intake were identified. These samples were marked as abnormal and used to validate the SHM approach since detection of abnormal patterns due to diet and drugs is in principle no different from detection of abnormalities related to a disease. Additionally, the set of 24 patients was used for validation. In all samples, the abnormal metabolites were identified by the clinical practitioner by comparison of the abnormal resonances to a database of NMR spectra of model compounds [5]. In cases where the overlap of resonances in the 1D spectra was quite severe, 2D COSY NMR experiments were used to provide additional information and confirm that the metabolite identification based on the 1D spectra was correct.

The SHM model was constructed on the basis of 120 training samples that were selected from the remaining set of 176 binned ^1^H NMR spectra of normal (healthy) individuals by the Kennard Stone algorithm [16]. The optimal number of factors in the PCA model was determined by a bootstrapping algorithm called NUMFACT [17]. In essence, the PCA factors determined for each resampling were compared for changes. Factors which changed significantly from one resampling to the next were probably due to noise and excluded from the model.

Validation of identification of abnormal metabolites was performed by applying the left-out 56 healthy; 17 healthy, but abnormal; and 25 patient samples to the SHM model. An imposed significance limit (*α*) of 5% was used. Note that centring and scaling of the test data was based on the feature means and standard deviations of the training data.

All analysis was performed using in-house developed algorithms. Bootstrapping to estimate the number of factors in PCA was performed with PLS_Toolbox 6.7.1 [18].

## Results

### Inspection of the ^1^H NMR data: current clinical practice

The data was first analysed according to current clinical practice, namely by visual inspection and by means of PCA scoreplots. This inspection was required to select the NOC samples (healthy individuals) on which the SHM model could be trained.

A clinical expert visually inspected the NMR data of the 193 healthy children and the 24 patients. Ten exogenous metabolites related to diet or drug intake were observed in the set of 193 children. More details are specified in Table 1. Seventeen samples contained metabolites related to bacteria, a fish meal, paracetamol, or cyclamate. The artificial sweeteners Acesulfame K and mannitol were present in the metabotype of such a large number of healthy individuals that they were not marked as abnormal metabolites. Fifteen abnormal metabolites were observed in the 24 patient samples. These were related to 7 IEM (18 patients) as well as medication (6 patients). More details are specified in Table 2.

**Figure 1:**
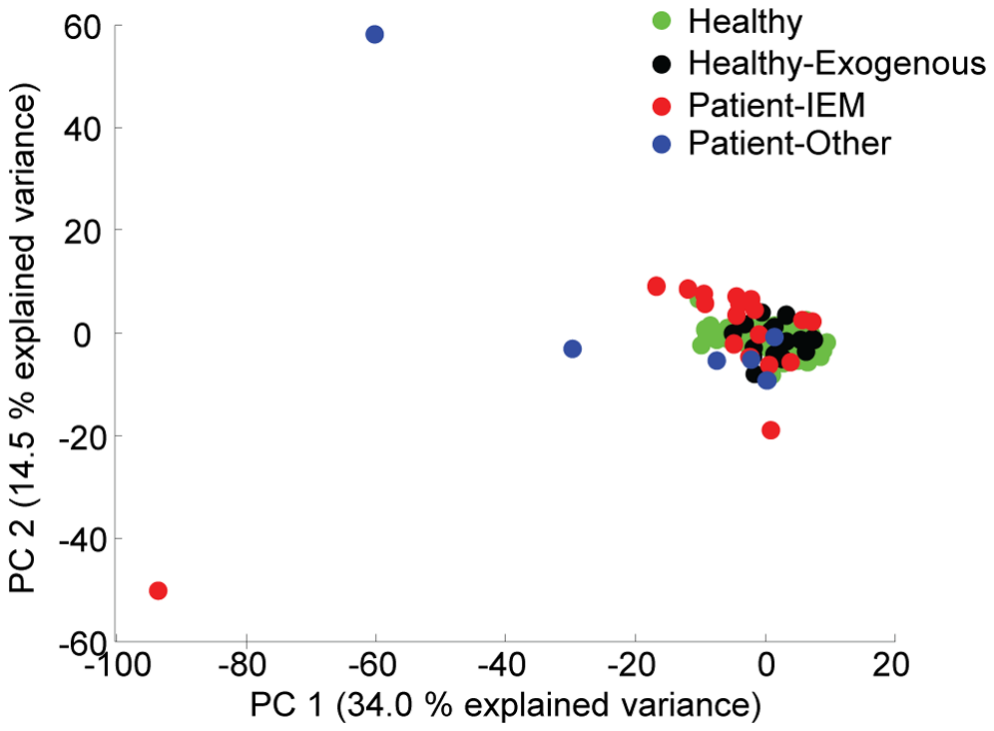
PCA scoreplot of the autoscaled data. Note that the points are coloured according to the observations made by a clinical expert as healthy; healthy, but exogenous metabolites were present; patients diagnosed with IEM; and other patients.

In figure 1, a PCA scoreplot of all samples is shown (autoscaled data). The samples were coloured according to the observations made by the clinical expert. Clearly, many abnormal metabotypes could not be distinguished from healthy samples this way. Alternative colourings of the plot indicated no trends related to age, gender or other demographics either.

### Statistical health monitoring

The SHM model was constructed on the basis of 120 healthy metabotypes. The clinical expert had not detected any of the exogenous metabolites listed in table 1 in these samples, except for the articial sweeteners Acesulfame and mannitol. This means that future samples that contain exogenous metabolites related to fish, paracetamol intake, etc will be marked as abnormal by the model even if they are healthy. This can be undesirable if the sole purpose of the SHM model is disease diagnosis. We will further elaborate upon this choice of NOC samples in the discussion.

**Figure 2:**
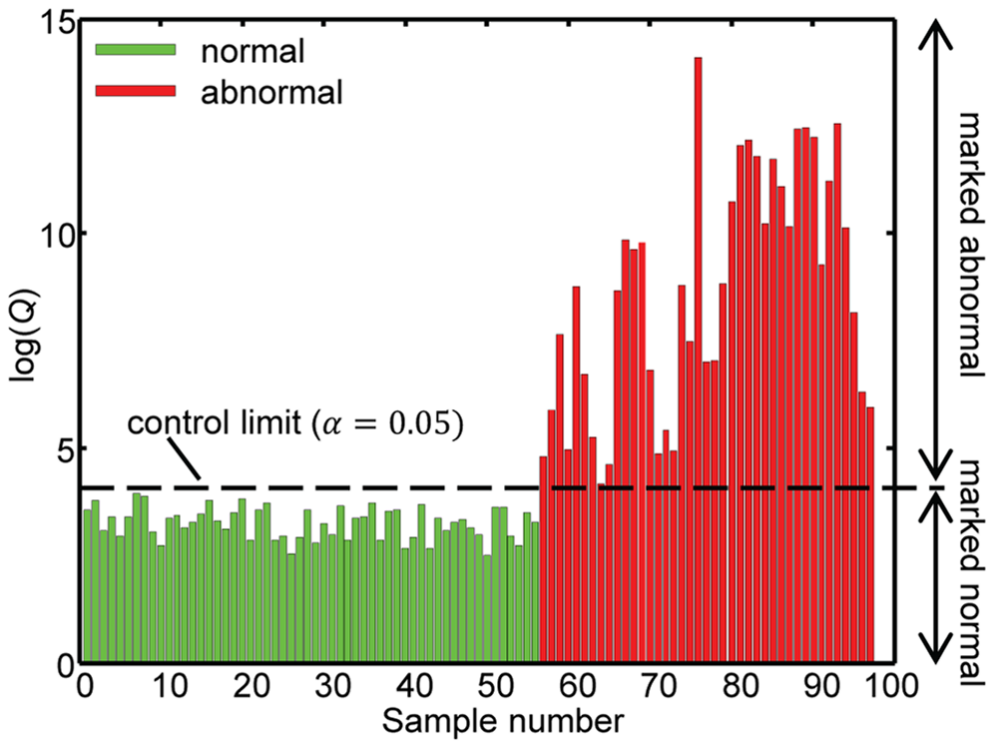
*Q*-values obtained by statistical health monitoring for 56 healthy and 42 abnormal metabotypes.

Eighty-three percent of the total variation in the NOC data was estimated to be systematic by NUMFACT. This variation was modelled by the first 16 factors. Next, the metabotype of the validation samples was automatically inspected using the constructed SHM model. As shown in figure 2, *Q*-values of the abnormal metabotypes were clearly larger compared to the normal metabotypes. Using the imposed significance limit of 5%, all normal and abnormal metabotypes were correctly identified. Note that the cut-off point *Q_5%_* to mark a patient's metabotype as abnormal was completely based on the training samples (equation 4).

The 17% variance left out of the model must correspond to individual variations, which did not necessarily belong to the NOC of the whole group. This unexplained variance partly re-appeared as *Q* contribution. Therefore, metabotypes of normal individuals did not have zero contribution for every feature. Statistically speaking, with the chosen significance level 5% of the samples that are within NOC are expected to be incorrectly marked as abnormal (i.e. 3 individuals). In this case all normal individuals were correctly detected which is related to size of our test cohort.

**Figure 3:**
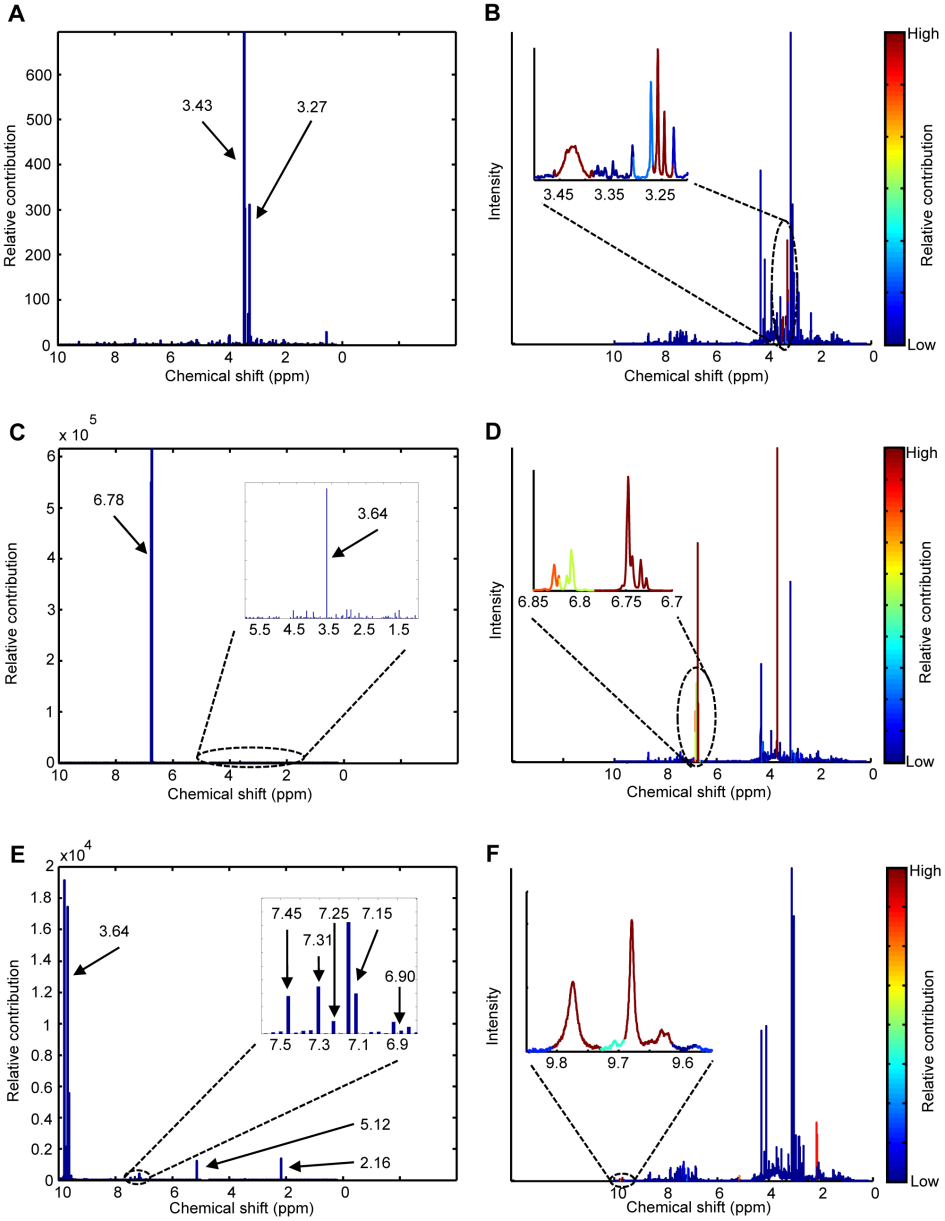
Feature contributions visualized in a contribution plot and the original NMR spectra for three abnormal metabotypes. The abnormal metabolites are related to (A, B) high concentrations of taurine, (C, D) alkaptonuria, and (D, E) paracetamol comsumption. The arrows indicate the resonance corresponding to the middle of a bin. Each bin had a width of 0.04 ppm.

For metabotypes marked as abnormal, the abnormal metabolites were identified via the relative feature contributions to *Q*. The set of relative contributions can be considered as a personal biomarker for that individual since they highlight how and how much this individual is different from NOC. The contribution can be visualized in a so-called contribution plot which is commonly done in industrial process control, or in the original NMR spectrum to integrate SHM in current clinical practice. In a contribution plot the relative contribution is plotted against the chemical shift value. Three examples are presented in figures 3a, c, and e. In each figure, high peaks relative to the baseline indicate resonances that were abnormal with respect to NOC. An advantage of contribution plots is that features with a large contribution are easy to identify, even if they have a low intensity in the original NMR spectrum (e.g. the resonances between 9.6–9.8 ppm in figures 3e and 3f). In contrast to contribution plots, visualization in the NMR spectrum itself allows the user to make combined use of contribution values as well as NMR knowledge such as multiplet structure to make a diagnosis. As shown in figures 3b, d, and f, the contribution values are colour coded in this type of visualization.

The abnormal metabotypes were further inspected using both representations of the relative contribution. All IEM, were correctly diagnosed this way. Similarly, all abnormalities related to diet and the different types of medication were correctly identified. Most IEM were diagnosed via key resonances – biomarkers relating to the specific IEM; not always were all relevant biomarker resonances for a specific IEM marked as abnormal. This is similar to visual inspection of the data: resonances with a high degree of splitting or overlap cannot be discerned from noise in 1D spectra.

In order to illustrate the principles of relative contributions and the procedure to follow to establish a potential diagnostic better, we will describe the three examples in figure 3 in more detail below. The complexity of these examples is progressively rising in terms of spectral interpretation meaning that correct identification by SHM is more challenging.

#### Case 1

The first example is considered relatively simple because only two resonances are involved. As shown in figure 3a, SHM clearly marked two resonances at 3.27 and 3.43 ppm as abnormal: the relative contribution was much larger compared to the contribution of other resonances. Similar to visual inspection of the data, the metabolite corresponding to these abnormal resonances was identified by comparison of the resonances to a database of NMR spectra of model compounds. These particular two resonances correspond to taurine indicating that the metabotype of this patient contained abnormally high concentrations of taurine. This was confirmed by visual inspection of the spectrum by the clinical expert. At the moment the cause of the high concentrations of taurine in the metabotype of this patient is unknown. Perhaps, the abnormality can be related to diet, e.g. consumption of energy drinks.

#### Case 2

The contribution plot of this patient also showed two resonances that were abnormal: a singlet at 3.64 ppm and a multiplet at 6.78 ppm (figures 3c and 3d). However, inspection of this plot was more difficult compared to case 1. The multiplet was much easier to detect compared to the singlet because the NMR spectra of healthy individuals did not contain much signal around 6.78 ppm. Therefore, the relative contributions of this multiplet were very large. In contrast, the singlet at 3.64 ppm was positioned in a crowded region of the spectra resulting in much lower contribution values. However, compared to the contributions at the surrounding chemical shifts, the singlet at 3.64 was still clearly abnormal. This shows that inspection of contribution plots should not only focus on the absolute value of the contributions, but on their size relative to the contribution that is observed for most chemical shifts. One could say that for each individual the “*Q* baseline” must be used to determine if a particular peak is abnormal or not. The abnormal singlet at 3.64 ppm and the multiplet at 6.78 ppm indicated that the metabotype of this individual contained a large concentration of homogentisic acid [5]. Thanks to this, the patient was diagnosed with the IEM alkaptonuria. Alkaptonuria is caused by a deficiency of the enzyme homogentisic acid oxidase in tyrosine catabolism [5]. This results in high concentrations of homogentisic acid in the urine of such a patient.

#### Case 3

As shown in figure 3e, eight regions in the NMR spectrum of this individual had abnormal contributions. Similar to case 2, the contributions of the relevant resonances again differed by orders of magnitude.

Comparison of the abnormal regions to spectra of model compounds clearly indicated that the metabolites acetaminophen, acetaminophen-glucuronide, and acetaminophen-sulphate were present in high concentrations (see table 1). This is caused by consumption of paracetamol by this individual. As shown in Table 1, this drug can actually be detected in urine via abnormal concentrations of five metabolites. A number of these metabolites will be visible in the NMR spectrum depending on how the drug was metabolized. In this case no high *Q*-values were observed at resonances 1.84, 6.99, and 7.51 ppm. This indicates that the compounds A-*N*-acetyl-*L*-cysteinyl and A-*L*-cysteinyl were either present in very low concentration, or that in this particular case paracetamol was not metabolised into these metabolites. This was confirmed by visual inspection of the NMR spectrum. Due to the large number of resonances involved, identification of paracetamol intake via SHM is considered more difficult compared to the previous two cases. Additionally, the intensities of the resonances involved are much lower which makes diagnosis even more difficult.

Note that for all individuals who consumed paracetamol, the resonances around 9.8 ppm were also marked as abnormal. These resonances have not been described in literature. However, by means of a simulated NMR spectrum of paracetamol in the Bruker software we ascribe these resonances to NH-groups in the molecule.

## Discussion

In this study, SHM was introduced as a valuable tool for diagnosis of a multitude of possible (rare) diseases. The method was successfully applied in a case study involving diagnosis of several IEM as well as metabolic abnormalities related to drug consumption and diet.

First, the metabotype of each individual was marked as normal or abnormal: 100% of the “healthy” and 100% of the abnormal metabotypes were correctly identified. Next, it was shown that feature contributions can be used to identify the abnormal metabolites. The contributions are very easy to calculate without prior knowledge. Prior knowledge, however, is required to interpret them and relate the abnormal features to a disease. Therefore, SHM should be regarded as a decision support tool for diagnosis. In case of rare diseases, SHM is the only tool available to detect the abnormalities. In case of more common diseases, the SHM-based metabotype screening could be followed by more classical targeted approaches (e.g. a two-class classifier) to confirm the diagnosis.

The first step of SHM, detection of abnormal metabotypes in a multivariate fashion, is functioning in a reliable way. The second step concerns identification of the abnormality. This identification relies on a univariate evaluation of the individual contributions of each feature or measured signal to *Q*. These contributions should be studied with some caution due to the so-called smearing effect: contributions from abnormal features can propagate to other features meaning that fault free features can show increased contribution [15]. This a well-known issue in industrial process control that has been greatly discussed in literature [13]. The smearing effect is a direct consequence of the fact that an SHM model is constructed on the basis of normal metabotypes. Because of this the model is very well able to detect when a metabotype is abnormal. However, when the abnormal metabolites are identified via the *Q*-statistic again information from the normal (NOC) individuals has to be used (see equation 7). The model assumes that the correlations between metabolites in the abnormal metabotype are the same as those encountered in the NOC samples. This doesn't have to be the case. This imperfect assumption may lead to some false positives i.e. some metabolites can be marked as abnormal while they are not. Unfortunately, the smearing effect cannot be avoided. In this study, the smearing effect was minimized by using partial decomposition of the *Q*-statistic, instead of the commonly used complete decomposition method. This ensures that the contribution of an abnormal feature will always be greater than the contribution given to the “good” feature [15].

Some works in the process control literature suggest the use of control limits for determining the significant feature contributions. However, this should be discouraged since the (biological) unrelated features might also show an increased contribution due to the smearing effect [15]. Therefore, we prefer to rely on human expertise by visually inspecting the contribution plots.

Selection of NOC samples is a critical step in the construction of an SHM model. The proposed method detects deviations from these normal samples. This deviation can be related to disease, but also a healthy sample with a deviation that is not present in the NOC set – e.g. the paracetamol example presented in case study 3. When disease diagnosis is the goal of the SHM model, examples such as case 3 are false positives. As shown in figure 1, the patient samples differed more from the NOC samples (the healthy group) compared to the group of healthy samples that contained exogenous metabolites. This was the main reason why the latter group was not included in NOC: we wanted to investigate if SHM was also able to detect these smaller deviations from NOC. This group should be included in NOC, however, if the user only wants to detect metabolites related to disease. Therefore, we also briefly investigated this disease diagnosis model. A direct consequence of the fact that the NOC now contained extra inter-individual variation due to diet and medication was that the cut-off value for *Q* (equation 4) was increased. This means that samples will less quickly be marked as abnormal, increasing the chance of false negatives. In this case, however, all patient samples were still correctly diagnosed. In contrast to the SHM model presented in the results section, all samples with metabolites related to medication and drugs were now marked as normal. In this feasibility study, the NOC set was a small population of healthy children. These samples matched in age and ancestry to the expected IEM patients. No restrictions on factors such as lifestyle were imposed to ensure enough diversity within the NOC set so that it is representative of future patient samples. However, due to the size of our cohort most likely not all possible factors such as all types of medication were included in NOC. We expect that a much larger cohort of randomly selected NOC samples would contain most of the common diets; types of medication; and other factors, thereby avoiding the occurrence of false positives related to this. Additionally, if false positives occur later on, the NOC set can be updated with these samples. Note that the cohort should match the expected demographics of the patients are closely as possible since the larger the biological variation of the NOC samples the more difficult it will be to detect a subtle abnormality related to disease.

When working with large cohorts of NOC samples, an interesting research line would be to see if sub-populations of normal individuals with completely different characteristics due to e.g. environmental factors can be identified. Each sub-population has its own NOC. In such a case, a SIMCA-like model structure where a separate SHM model is constructed for each population seems more appropriate compared to one general model that was used in the present study. Matching new samples only against NOC of the correct sub-group could greatly enhance the power of the SHM model for disease diagnosis. If the subpopulations are unknown, perhaps a clustering approach such as mixture modelling can be used to define them.

Three additional future development lines can be defined for SHM: (1) connection of SHM output to a disease database, (2) development of personalized health control, and (3) application of SHM in clinical trials. The first research line could be implemented in SHM in the form of a database of known disease which would automatically link the abnormalities detected with a list of potential diseases.

The second perspective is to define NOC at an individual level instead of a population or sub-group based one. To do so, longitudinal metabotyping experiments should be performed. The SHM model would then very precisely describe the NOC metabotype profile because no intra-individual differences have to be taken into account. In consequence, SHM would be able to detect more subtle abnormalities. An additional advantage of longitudinal studies for detection of abnormal metabotypes is that the user can accumulate information from a series of measurements. One could check whether measurements appear randomly distributed between the control limits or if a structure is appearing, signalling the start of a deviation from NOC. Such tests may greatly improve the power of SHM for disease detection. Identification of the specific abnormality may be improved by studying contributions relative to the last *k* timepoints instead of all NOC samples. The abnormal metabotype should be most similar to the last metabotypes that were measured before the individual became ill.

In this study, NMR was used to assess the metabotype of each individual because it is a very stable technique with a detection limit in the low micromolar range. This technique has been used routinely for over 20 years in Radboud University Medical Centre in Nijmegen to diagnose IEM. Although NMR is a valuable analytical platform to diagnose IEM, it is not necessarily the best technique for other diseases. Other data types such as results of classical blood tests or more advanced measurements such as 2D-NMR and LC-MS should be used if they are known to provide more relevant information. In principle, SHM can be applied to any type of data. For each application it is important to take into account the structure of the data and adjust the model accordingly. Here, PCA was used to describe healthy metabotypes. Multiway data coming from 2D-NMR or LC-MS could be evaluated using a PARAFAC or Tucker3 structure [19]. Because SHM can be applied to any data type, it will most likely not only find application in metabolomics, but also in other fields such as proteomics or genomics.

## Conclusion

Due to the complex nature of metabolomics data, multivariate statistics are required interpret the data. Unfortunately, current multivariate tools can only diagnose diseases in a targeted fashion; a separate model is required for each disease. Additionally, such tools are not always applicable to rare or orphan diseases. Abnormal metabotypes can sometimes be detected in an untargeted fashion by visual comparison of the data. However, detection of subtle abnormalities and abnormal patterns is extremely subjective and time-consuming. An alternative approach, SHM, was proposed in this study.

In SHM, the metabotype of an individual is compared to normal (healthy) metabotypes in a multivariate manner. Any abnormal patterns are indicated by the method. Subsequently, this information can be used for diagnosis. In this study, SHM was successfully applied for diagnosis of various metabotypic abnormalities related to diet, drug intake and IEM.

SHM is a general method that is not only applicable to metabolomics data. Additionally, the method offers perspectives in the framework of personalized health.

## Supporting Information

File S1 **An example of SHM when monitoring 2 metabolites is presented as supporting material.**
(DOCX)
