# Correction: Towards the Disease Biomarker in an Individual Patient Using Statistical Health Monitoring

**DOI:** 10.1371/journal.pone.0097371

**Published:** 2014-05-05

**Authors:** The PLOS ONE Staff

## Notice of Republication

This article was republished on April 16, 2014, to correct errors in the numbering of equations that were introduced during the production process. The publisher apologizes for the errors. Please download this article again to view the correct version.
