# Supplementary material for: Towards the Disease Biomarker in an Individual Patient Using Statistical Health Monitoring
Source: PLoS One. 2014 Apr 1;9(4):e92452. doi: 10.1371/journal.pone.0092452 (PMC3972152; doi:10.1371/journal.pone.0092452)
Supplement: File S1 — An example of SHM when monitoring 2 metabolites is presented as supporting material. (DOCX) [file pone.0092452.s001.docx]

**SUPPORTING INFORMATION 1**

In figure S1, disease detection is illustrated for a problem where 2 metabolites are monitored. Note that in comparison to the paper, a second statistic (*T^2^*) is show here as well. This statistic is often used in statistical process control to monitor the values of the scores of the PCA model. In our application this statistic does not offer any additional information compared to the *Q*-statistic. However, this statistic can be useful when SHM is applied to other types of data.

Figure S1 shows a a 1-factor (or principal component) PCA model that describes the main differences between the NOC samples. Outlier 1 has a low value for the *Q*-statistic, but a high value for the *T^2^*-statistic. The metabotype of this individual is explained well by the model but is different from the NOC metabotypes in the sense that it is an extreme object (it has an extreme score). This means that the sample has extreme metabolite concentrations, but the overall between-metabolite correlation pattern is conserved, e.g. due to improper correction for dilution effects. Outliers 2 and 3 have a high value for the *Q*-statistic. The metabotype of these individuals is not described well by the model because the relation between the two metabolite concentrations is very different from NOC. In other words, the *Q*-statistic can detect abnormal correlations between the metabolites. Therefore this statistic is the most suitable for disease diagnosis.


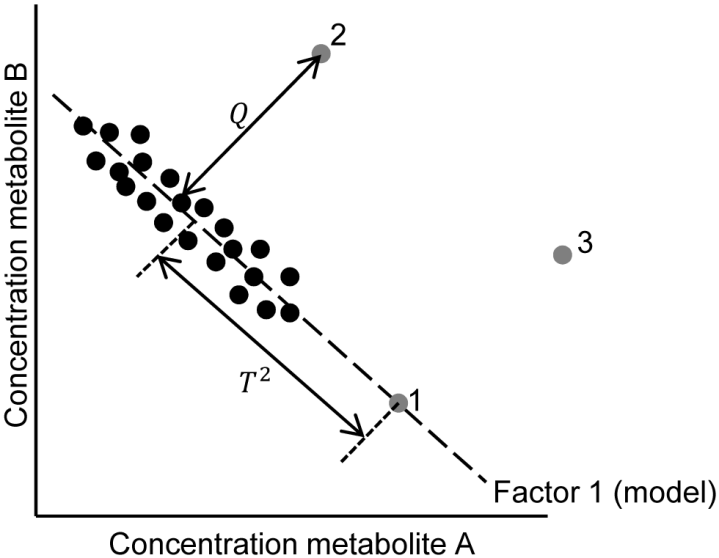


**Figure S1. Disease detection via statistical health monitoring for a two metabolites problem.**

The black dots indicate NOC samples. The grey dots are outliers.
